# 非小细胞肺癌脑转移放疗时机的选择及疗效预后分析

**DOI:** 10.3779/j.issn.1009-3419.2016.08.04

**Published:** 2016-08-20

**Authors:** 洋 王, 健 方, 鋆 聂, 玲 戴, 维亨 胡, 洁 张, 向娟 马, 金娣 韩, 筱玲 陈, 广明 田, 頔 吴, 森 韩, 皆然 龙

**Affiliations:** 100142 北京, 北京大学肿瘤医院暨北京市肿瘤防治研究所, 恶性肿瘤发病机制及转化研究教育部重点实验室, 胸部肿瘤内二科 Key Laboratory of Carcinogenesis and Translational Research (Ministry of Education), the Second Department of Thoracic Oncology, Peking University Cancer Hospital and Institute, Beijing 100142, China

**Keywords:** 肺肿瘤, 脑转移, 脑放疗, 化疗, 靶向治疗, Lung neoplasms, Brain metastases, Brain radiotherapy, Chemotherapy, Targeted therapy

## Abstract

**背景与目的:**

放疗联合化疗或靶向治疗仍然是非小细胞肺癌脑转移患者的首选治疗。本研究旨在探讨脑放疗时机的选择及推迟脑放疗对于患者疗效和生存期的影响。

**方法:**

2003年5月-2015年12月, 就诊于我中心确诊为非小细胞肺癌脑转移且接受过脑放疗及全身治疗(包括化疗和靶向治疗)的患者共198例入组了本研究。127例接受了同时性的全身治疗和脑放疗(早放疗组)。71例患者接受了延迟性的脑放疗:完成至少2周期全身治疗后才接受脑放疗(晚放疗组)。早放疗组和晚放疗组患者治疗前的脑转移预后评分(DS-GPA评分)均衡无偏倚。

**结果:**

早放疗组患者的中位生存时间(overall survival, OS)与晚放疗组相比明显延长(17.9个月 *vs* 12.6个月, *P*=0.038), 早放疗组患者的无进展生存期(progression-free survival, PFS)也优于晚放疗组(4.0个月 *vs* 3.0个月, *P* < 0.01)。3级-4级放化疗不良反应的发生率两组间无明显差异。确诊脑转移后, 一线使用酪氨酸激酶抑制剂(tyrosine kinase inhibitor, TKI)药物可能延长患者的OS(17.9个月 *vs* 15.2个月, *P*=0.289), 但无明显统计学差异。在整个疾病进展过程中曾经使用TKI类药物与从未使用过TKI药物相比, 患者的OS延长(20.0个月 *vs* 10.7个月, *P* < 0.01)。

**结论:**

对于非小细胞肺癌脑转移患者, 推迟脑放疗可能会影响患者的生存期。这一发现仍需要前瞻性多中心的随机对照研究来证实。

## Abstract (en)

**Background and objective:**

Radiotherapy combined with chemotherapy or molecular targeted therapy remains the standard of treatment for brain metastases from non-small cell lung cancer (NSCLC).The aim of this study is to determine if the deferral of brain radiotherapy impacts patient outcomes.

**Methods:**

Between May 2003 and December 2015, a total of 198 patients with brain metastases from NSCLC who received both brain radiotherapy and systemic therapy (chemotherapy or targeted therapy) were identified.The rate of grade 3-4 adverse reactions related to chemotherapy and radiotherapy had no significant difference between two groups.127 patients received concurrent brain radiotherapy and systemic therapy, and 71 patients received deferred brain radiotherapy after at least two cycles of chemotherapy or targeted therapy.Disease specific-graded prognostic assessment was similar in early radiotherapy group and deferred radiotherapy group.

**Results:**

Median overall survival (OS) was longer in early radiotherapy group compared to deferred radiotherapy group (17.9 months *vs* 12.6 months; *P*=0.038).Progression free survival (PFS) was also improved in patients receiving early radiotherapy compared to those receiving deferred radiotherapy (4.0 months *vs* 3.0 months; *P* < 0.01).Receiving tyrosine kinase inhibitor (TKI) therapy after the diagnosis of brain metastases as any line therapy improved the OS (20.0 months *vs* 10.7 months; *P* < 0.01), whereas receiving TKI as first line therapy did not (17.9 months *vs* 15.2 months; *P*=0.289).

**Conclusion:**

Our study suggests that the use of deferred brain radiotherapy may resulted in inferior OS in patients with NSCLC who develop brain metastases.A prospective multi-central randomized study is imminently needed.

肺癌是发病率及死亡率最高的肿瘤, 其中非小细胞肺癌(non-small cell lung cancer, NSCLC)占多数。大约20%-40%的NSCLC患者在疾病的进程中都会发生脑转移^[1, 2]^。NSCLC脑转移的患者预后很差, 自然状态下中位生存期只有3.4个月^[3]^。近年来, 随着放疗技术的飞速发展, 系统性全身治疗的日臻完善规范, 特别是小分子靶向药物的广泛应用, NSCLC脑转移患者的预后有了极大的改善^[4, 5]^。多项研究^[6, 7]^表明化疗或靶向治疗联合脑放疗, 对比单独脑放疗, 有着更好的协同效应, 对脑转移有着更高的控制率, 并且能够延长患者的生存。但是由于脑放疗特别是全脑放疗对神经认知功能可能造成损害, 并且放疗和全身治疗同时进行可能加重毒性反应, 有一部分研究认为对于脑转移患者, 可以先行全身治疗, 待脑转移病灶进展后再行脑放疗^[8]^。因此, 脑放疗时机的选择, 仍然存在争议。本研究回顾性分析了接受脑放疗和全身治疗的NSCLC脑转移患者中, 放疗时机的选择对患者肿瘤控制率、安全性、生存期的影响。

## 1 资料和方法

### 1.1 临床资料

本研究收集了2003年5月-2015年12月北京肿瘤医院胸内二科收治的NSCLC脑转移患者, 入组标准包括:经病理或组织学确诊为NSCLC, 经头颅磁共振成像(magnetic resonance imaging, MRI)或计算机断层扫描(computed tomography, CT)证实有脑转移, 先后或同时接受过全身治疗(包括化疗和靶向治疗)及放疗, 可评价疗效。经过筛选, 共有198例患者入组本研究。我们收集了患者的一般临床资料:包括年龄、性别、体力状况评分(Eastern Cooperative Oncology Group, ECOG)、吸烟状况、脑转移时间、是否初治时发现脑转移、脑转移个数、脑转移症状、颅外转移情况等, 同时我们采用GPA预后评分系统(graded prognostic assessment)对脑转移患者进行了预后评分。

### 1.2 治疗方法

所有的患者均接受了全身治疗(包括化疗和靶向治疗)和脑放疗。患者确诊为NSCLC脑转移后, 我们将全身治疗开始2个周期内接受脑放疗的患者定义为早放疗组, 将全身治疗结束后或全身治疗2个周期后接受脑放疗的患者定义为晚放疗组。确诊脑转移后首次全身治疗为一线治疗的病例有170例, 二线治疗的病例有15例, 三线治疗的病例有3例。确诊脑转移后首次全身治疗的方案包括:含铂双药方案155例(吉西他滨联合顺铂或奈达铂110例, 培美曲塞联合顺铂或卡铂27例, 长春瑞滨联合顺铂9例, 多西他赛联合顺铂5例, 紫杉醇联合顺铂4例), 靶向药物37例(吉非替尼20例, 埃克替尼10例, 厄洛替尼6例, 克唑替尼1例), 单药化疗6例(多西他赛3例, 吉西他滨2例, 培美曲塞1例)。有125例患者在疾病治疗过程中曾经使用过靶向药物, 其中阿帕替尼4例, 克唑替尼1例, 余均为一代表皮生长因子受体酪氨酸激酶抑制剂(epidermal growth factor receptor tyrosine kinase inhibitors, EGFR-TKI)(吉非替尼、厄洛替尼、埃克替尼)。放疗采用全脑放疗(whole-brain radiotherapy, WBRT)或立体定向放射外科(stereotactic radiosurgery, SRS)。其中175例患者仅接受了WBRT, 7例患者仅接受SRS, 16例患者先后接受WBRT和SRS。另外有3例患者曾接受头颅手术治疗, 后均接受了WBRT治疗。WBRT的剂量为30 Gy/10 f或40 Gy/20 f, SRS的剂量为15 Gy -24 Gy。

### 1.3 疗效及毒性评价

疗效评价采用实体瘤疗效评价标准(Response Evaluation Criteria in Solid Tumors, RECIST)1.1版。总生存期(overall survival, OS)定义为从确诊脑转移至死亡或末次随访。无进展生存期((progression-free survival, PFS)定义为从确诊脑转移后首次治疗到疾病进展或死亡。毒性评价采用美国国立癌症研究所常用药物毒性反应标准(Common Toxicity Criteria, CTC)4.0版。

### 1.4 统计学方法

采用SPSS 22.0统计软件进行统计分析。计数资料组间差异比较采用卡方检验或Fisher精确检验, 生存率分析采用*Kaplan-Meier*法, 生存率的组间比较采用*Log-rank*检验。*P* < 0.05为差异有统计学意义。

## 2 结果

### 2.1 临床资料特点

入组本研究的NSCLC脑转移患者临床资料如表 1所示。大部分病例为男性(57.1%), 小于60岁(69.2%), ECOG评分0分-1分(90.9%), 其中腺癌占绝大多数(86.9%), 其次为鳞癌(8.6%)。91.4%的患者确诊为肺癌时为Ⅳ期, 78.8%的患者初次诊断时就已经有脑转移, 79.3%的患者同时存在颅外转移。脑转移有临床症状的占31.3%。根据GPA预后评分系统, 预后较差的0分-2分占多数(70.2%)。127例接受了同时性的全身治疗和脑放疗(早放疗组), 71例患者接受了延迟性的脑放疗(晚放疗组)。早放疗组和晚放疗组在年龄、性别、ECOG评分、病理类型、脑转移个数、颅外转移情况、GPA评分方面无明显统计学差异。

**1:** 非小细胞肺癌脑转移患者临床特征 Clinical characteristics of NSCLC patients with brain metastases

Characteristic	Early radiotherapy group (*n*=127)	Deferred radiotherapy group (*n*=71)	Total	*P*-value
Age, years				0.356
≤60	85 (66.9%)	52 (73.2%)	137 (69.2%)	
> 60	42 (33.1%)	19 (26.8%)	61 (30.8%)	
Gender				0.176
Male	77 (60.6%)	36(50.7%)	113 (57.1%)	
Female	50 (39.4%)	35 (49.3%)	85 (42.9%)	
Smoking				0.044
Former/Current	56 (44.1%)	21 (29.6%)	77 (38.9%)	
Never	71 (55.9%)	50 (70.4%)	121 (61.1%)	
ECOG				0.117
0	67 (52.8%)	29 (40.8%)	96 (48.5%)	
1	47 (37.0%)	37 (52.1%)	84 (42.4%)	
2	13 (10.2%)	5 (7%)	18 (9.1%)	
Histologic type				0.819
Adenocarcinoma	110 (86.6%)	62 (87.3%)	172 (86.9%)	
Squamous carcinoma	10 (7.9%)	7 (9.9%)	17 (8.6%)	
Adenosquamous	2 (1.6%)	1 (1.4%)	3 (1.5%)	
Large cell carcinoma	2 (1.6%)	0	2 (1%)	
Unidentified	3 (2.4%)	1(1.4%)	4 (2%)	
Stage at diagnosis				0.633
Ⅰ-Ⅲ	10 (7.9%)	7 (9.1%)	17 (8.6%)	
Ⅳ	117 (92.1%)	64 (90.1%)	181 (91.4%)	
Graded prognostic assessment				0.960
0-2.0	89 (70.1%)	50 (70.4%)	139 (70.2%)	
2.5-4.0	38(29.9%)	21 (29.6%)	59 (29.8%)	
Symptomatic from brain metastasis				0.176
Yes	44 (34.6%)	18 (25.4%)	62 (31.3%)	
No	83 (65.4%)	53 (74.6%)	136 (68.7%)	
Number of brain metastasis				0.177
1	43 (33.9%)	27 (38%)	70 (35.4%)	
2-3	52 (40.9%)	20 (28.2%)	72 (36.4%)	
≥4	32 (25.2%)	24(33.8%)	56 (28.3%)	
Extracranial metastasis present				0.365
Yes	98 (77.2%)	59 (83.1%)	157 (79.3%)	
No	29 (22.8%)	12 (16.9%)	41 (20.7%)	
NSCLC:non-small cell lung cancer; ECOG:Eastern Cooperative Oncology Group.				

### 2.2 疗效评价

随访采用电话随访或门诊回访, 随访至2016年5月, 随访率为94%, 中位随访时间为49个月。早放疗组的客观有效率(objective response rate, ORR)稍优于晚放疗组, 但并未达到统计学差异(25.2% *vs* 16.9%;*P*=0.178)。两组的疾病控制率(disease control rate, DCR)无明显差异(78.0% *vs* 76.1%;*P*=0.76)。对于脑转移治疗后初次进展部位, 两组间有明显差异(*P*=0.039, 表 2), 早放疗组倾向于先出现颅外转移。两组的PFS和OS也有明显的差别(图 1A, 图 1B)。早放疗组的PFS(4.0个月 *vs* 3.0个月, *P* < 0.01)和OS(17.9个月 *vs* 12.6个月, *P*=0.038)都明显优于晚放疗组。

**2:** 患者初始进展部位 Site of first progression in patients

Site of first progression	Early radiotherapy group (*n*=127)	Deferred radiotherapy group (*n*=71)	*P*
Intra-cranial	37 (29.1%)	31 (43.7%)	0.039
Extra-cranial	90 (70.9%)	40 (56.3%)	

**1:**
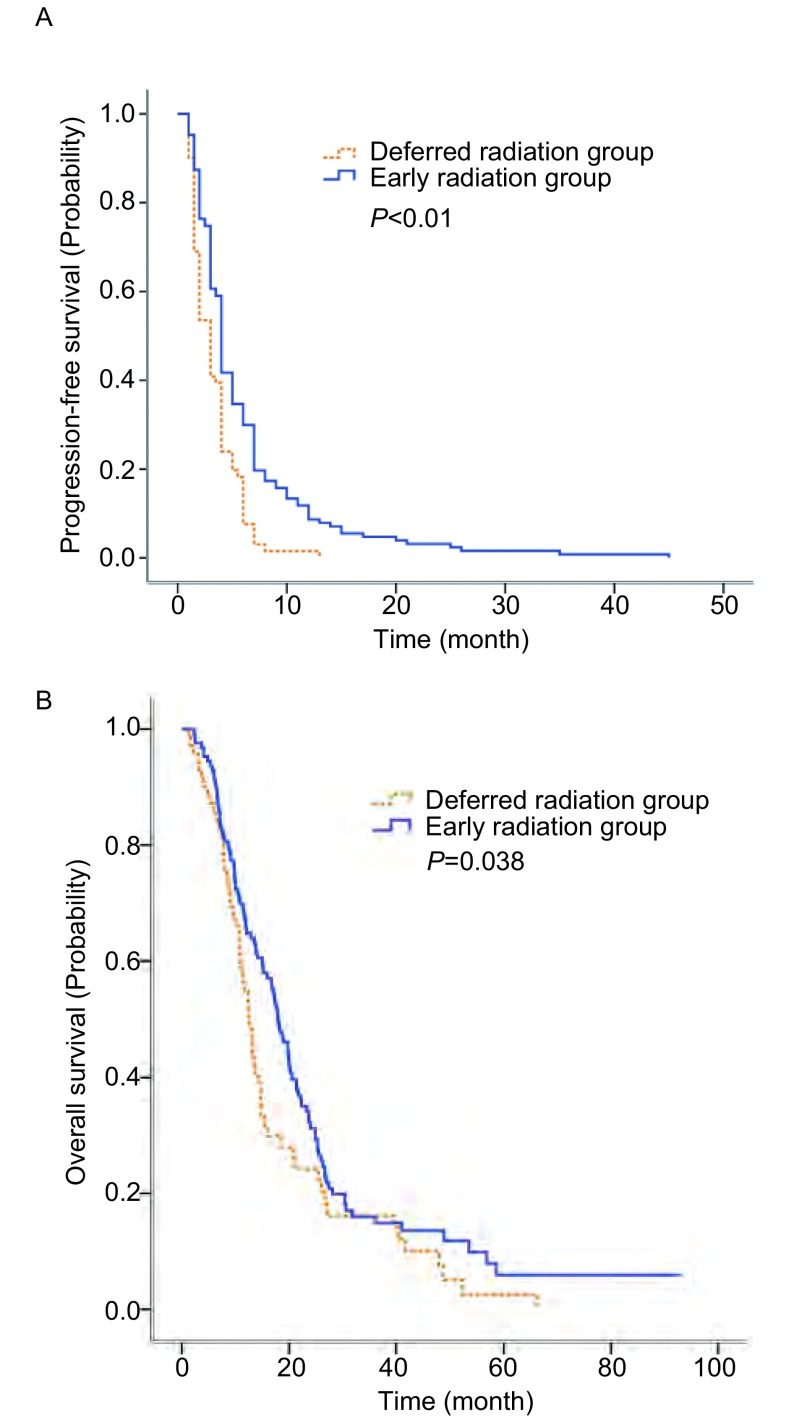
早放疗组与晚放疗组生存曲线(*Kaplan-Meier*法)。A:无进展生存期; B:总生存期。 *Kaplan-Meier* estimate of (A) progression-free survival and (B) overall survival, stratified by early radiation group or deferred radiation group.

### 2.3 靶向药物对疗效的影响

确诊脑转移后一线使用TKI类药物与一线使用化疗相比可提高患者的PFS(5.0个月 *vs* 3.0个月; *P* < 0.01), 但是对生存期(图 2A)的提高无明显统计学意义(17.9个月 *vs* 15.2个月; *P*=0.289)。确诊脑转移后任意线曾使用靶向药治疗的患者, 相比从未使用靶向药的患者, 有生存获益(图 2B), 且差异有统计学意义(20.0个月 *vs* 10.7个月; *P* < 0.01)。

**2:**
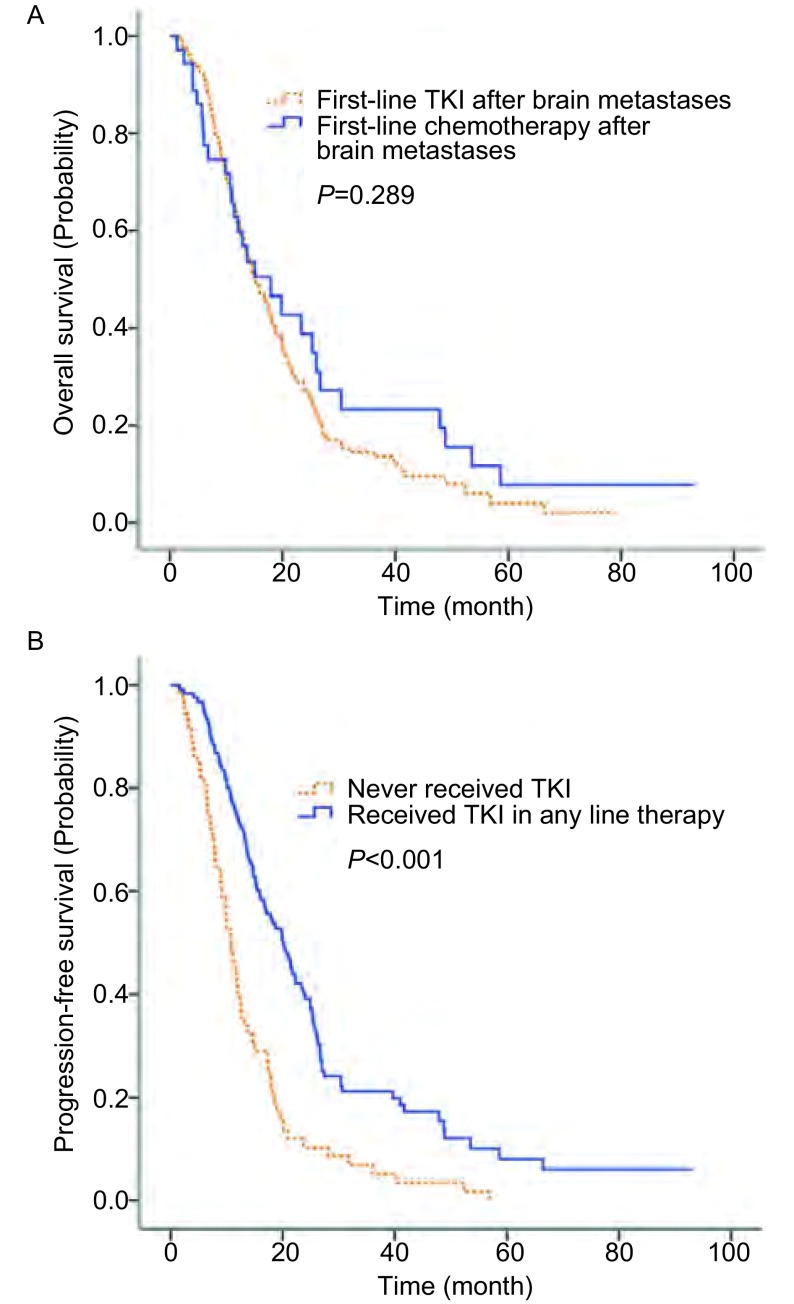
TKI的使用对生存期的影响。A:确诊脑转移后一线TKI组与一线化疗组生存曲线(*Kaplan-Meier*法); B:确诊脑转移后使用过TKI组与未使用过TKI组生存曲线(*Kaplan-Meier*法)。 Impact of TKI treatment on OS.A:*Kaplan-Meier* estimate of OS stratified by whether using TKI as first line therapy from the date of diagnosis of brain metastases.B:*Kaplan-Meier* estimate of OS stratified by whether using TKI as any line therapy from the date of diagnosis of brain metastases.OS:overall survival; TKI:tyrosine kinase inhibitors.

### 2.4 毒性评价

我们评价了常见的一些放化疗后毒性反应, 包括血液学毒性、消化道反应、乏力等(表 3)。大部分患者为1级-2级的毒性反应。3级-4级的毒性反应早放疗组和晚放疗组无明显的统计学差异。

**3:** 主要不良反应 Summary of the mostly commonly reported adverse events

Adverse event	Early radiotherapy group (*n*=127)	Deferred radiotherapy group (*n*=71)	*P*
Neutropenia			0.226
Grade 1/2	58	24	
Grade 3/4	12	14	
Thrombocytopenia			0.129
Grade 1/2	8	3	
Grade 3/4	11	6	
Anemia			0.093
Grade 1/2	20	7	
Grade 3/4	2	3	
Nausea & vomiting			0.326
Grade 1/2	63	26	
Grade 3/4	6	3	
Fatigue			0.077
Grade 1/2	20	5	
Grade 3/4	0	1	

## 3 讨论

脑转移是NSCLC晚期的重要表现, 是疾病进展和死亡的重要原因。本研究入组患者的中位生存期为13.7个月(1.6个月-93个月), 与既往报道的生存期相符^[9]^。虽然早放疗组的客观缓解率和DCR并未明显优于晚放疗组, 但是早放疗组的PFS和OS都明显优于晚放疗组(PFS 4.0个月 *vs* 3.0个月, *P* < 0.01;OS 17.9个月 *vs* 12.6个月, *P*=0.038)。两组患者的DS-GPA预后评分、ECGO评分、颅外转移情况、后续化疗情况、TKI类药物使用情况都是均衡的, 因此早放疗组生存期的延长, 可能是因为早放疗对脑转移更好的控制率导致, 而并非是由于选择偏倚导致的。早放疗组倾向于先出现颅外转移, 且有明显的统计学差异, 因此颅内转移的控制, 对于提高NSCLC脑转移患者的PFS, 延长患者的OS, 都有着至关重要的作用。

不良反应方面, 早放疗组和晚放疗组的不良反应都多为1级-2级不良反应, 大部分可耐受, 3级-4级不良反应的发生率两组间无统计学差异, 证实了尽早开始放疗并未提高放化疗不良反应的发生率。但是由于脑放疗造成的神经认知功能损害与脑转移进展后症状很难鉴别, 我们并未统计神经认知功能损害的不良反应发生率。

最近耶鲁大学的一项回顾性临床研究^[10]^表明:对于*EGFR*突变型的NSCLC脑转移患者, 先接受脑放疗后再接受全身EGFR-TKI靶向治疗, OS和颅内PFS都明显的优于先接受靶向治疗后再接受脑放疗的患者(OS 34.1个月 *vs* 19.4个月, *P*=0.005)。特别是先接受SRS放疗再接受靶向治疗的这一组患者, 中位生存期可达到58.4个月。但是也有研究^[8]^表明, 一线接受脑放疗, 对于无脑转移症状的*EGFR*突变型NSCLC脑转移患者, 并不能延长他们的PFS和OS。目前有一个在研的Ⅱ期临床研究对比厄洛替尼同步联合WBRT和厄洛替尼治疗直至脑转移进展后再行WBRT在*EGFR*突变型的NSCLC脑转移患者中的疗效预后分析(clinicaltrails.gov NTC01763385)。期待这项前瞻性研究能对这一问题的解决带来新的曙光。

本研究中, NSCLC脑转移的患者, 一线使用靶向药物虽未带来生存获益, 但是在疾病进展过程中曾经使用靶向药物明显延长了患者的生存时间。由于本研究中大部分接受靶向治疗的患者都为EGFR-TKI治疗(96%), 我们重点探讨TKI类药物对脑转移的治疗。多项研究^[11, 12]^表明, *EGFR*突变型的脑转移患者相比EGFR野生型的患者对脑放疗敏感性更高。同时有研究表明对于*EGFR*突变型的NSCLC脑转移患者, 接受EGFR-TKI治疗有着较高的客观有效率(70%-89%), 且可以延长患者的OS和PFS(OS 12.9个月-19.8个月和PFS 6.6个月-23.3个月)^[13, 14]^。本研究中虽未检测*EGFR*突变情况, 但入组的患者大部分为腺癌(86.9%), 且在亚裔腺癌患者中*EGFR*突变率较高。因此, 本研究中EGFR-TKI药物对NSCLC脑转移患者生存期的延长, 可能还是由于*EGFR*突变型的脑转移患者对EGFR-TKI反应较好, 这需要进一步的研究来细化患者的*EGFR*突变情况。虽然本研究中只有1例使用克唑替尼的患者, 但是最近的研究表明, 对于ALK阳性的NSCLC脑转移患者, 接受ALK抑制剂治疗, 中位生存期可以达到49.5个月, 颅内病灶的PFS也可达到11.9个月^[4]^。在个体化多靶点的精准治疗时代, 靶向治疗对于脑转移显示出了良好的疗效, 未来需要更多的临床试验去进一步探索和证实。

本研究仍然存在很多不足。首先, 本研究是一项回顾性研究, 有一定的局限性。其次, 本研究入组的患者, 大部分接受的是WBRT, 只有少数接受了SRS。WBRT对神经认知功能造成的损伤^[15, 16]^, 特别是对短时记忆的影响一直是人们关注的问题。遗憾的是, WBRT造成的认知功能损害很难准确界定。90%的脑转移病例在基线状态或WBRT前都存在一项以上认知功能损害。一项来自日本的研究^[17]^表明, 仅接受SRS放疗的患者简易智能量表评分明显下降, 该研究得出神经认知功能的损害大部分是由脑转移造成的, 而并非由脑放疗造成的。脑转移病灶的良好控制, 有可能带来患者认知功能的获益。

综上所述, 对于NSCLC脑转移患者, 在全身化疗或靶向治疗的同时, 尽早的开始脑放疗, 有助于提高颅内转移控制率, 改善预后, 延长患者生存时间。对于脑放疗时机的选择, 大规模随机前瞻对照性临床试验亟待开展。
